# Clinical effectiveness of beta-lactams versus fluoroquinolones as empirical therapy in patients with diabetes mellitus hospitalized for urinary tract infections: A retrospective cohort study

**DOI:** 10.1371/journal.pone.0266416

**Published:** 2022-03-31

**Authors:** Yu-Hsin Tang, Po-Liang Lu, Ho-Yin Huang, Ying-Chi Lin

**Affiliations:** 1 Master Program of Clinical Pharmacy, School of Pharmacy, College of Pharmacy, Kaohsiung Medical University, Kaohsiung, Taiwan; 2 School of Pharmacy, College of Pharmacy, Kaohsiung Medical University, Kaohsiung, Taiwan; 3 School of Post-Baccalaureate Medicine, College of Medicine, Kaohsiung Medical University, Kaohsiung, Taiwan; 4 Division of Infectious Disease, Department of Internal Medicine, Kaohsiung Medical University Hospital, Kaohsiung Medical University, Kaohsiung, Taiwan; 5 Center for Liquid Biopsy and Cohort Research, Kaohsiung Medical University, Kaohsiung, Taiwan; 6 Department of Pharmacy, Kaohsiung Medical University Hospital, Kaohsiung Medical University, Kaohsiung, Taiwan; 7 Master Degree Program in Toxicology, College of Pharmacy, Kaohsiung Medical University, Kaohsiung, Taiwan; 8 Doctoral Degree Program in Toxicology, College of Pharmacy, Kaohsiung Medical University, Kaohsiung, Taiwan; Medical University of Gdansk, POLAND

## Abstract

**Background:**

Diabetic patients are at risk of severe urinary tract infections (UTIs). Due to the emerging resistance rates to fluoroquinolones and β-lactams, we aimed to evaluate the effectiveness of β-lactams versus fluoroquinolones as empirical therapy for diabetic patients hospitalized for UTIs.

**Methods:**

A retrospective cohort study was conducted in a medical center in Taiwan between 2016 and 2018. Patients with type 2 diabetes, aged ≥20 and hospitalized for UTIs were enrolled. Patients with UTI diagnosis within one year before the admission, co-infections at the admission, or ≥2 pathogens in the urine cultures were excluded. The primary outcome was empiric treatment failure.

**Results:**

298 patients were followed for at least 30 days after the admission. *Escherichia coli* (61.07%) was the most common pathogen. The resistance rates of the pathogens to levofloxacin were 28.52% and 34.22% according to the historical Clinical and Laboratory Standards Institute (CLSI) breakpoints and the updated 2019 CLSI breakpoints, respectively. The resistance rates of ceftazidime and cefepime were 21.81% and 11.41%, respectively. Empirical β-lactams were associated with less treatment failure compared to fluoroquinolones (adjusted OR = 0.32, 95% CI = 0.17–0.60). Beta-lactams were associated with less treatment failure than fluoroquinolones when appropriatness was determined by the pre-2019 CLSI breakpoints but not the 2019 CLSI breakpoints.

**Conclusions:**

In diabetic patients hospitalized for UTIs, β-lactams were associated with less empiric treatment failure compared to fluoroquinolones when the resistance rate to fluoroquinolone is higher than β-lactams. The updated 2019 CLSI breakpoint for fluoroquinolone was better than pre-2019 CLSI breakpoints to correlate with treatment outcomes for hospitalized UTIs in diabetic patients.

## Introduction

Urinary tract infections (UTIs) were estimated to affect about 150 million patients worldwide and resulting in more than six billion dollars in healthcare expenditures every year [1]. Diabetes has been associated with UTIs which are difficult to treat, more likely to recur, and increased morbidity and mortality [2]. UTIs in diabetic patients are regarded as complicated UTIs that effective antimicrobial therapy is very necessary [3, 4].

Fluoroquinolones and β-lactams are common empirical antimicrobial choices for patients with UTIs requiring hospitalization [5, 6]. The prevalence of fluoroquinolone-resistant *Escherichia coli* from UTIs was approximately 25% in the USA and fluoroquinolone-resistant Gram-negative urinary isolates were >40% in the Asia-Pacific region [7–10]. However, ciprofloxacin and levofloxacin are primarily excreted via urine so they can reach very high concentrations in urine so that these agents may still be effective against UTIs caused by fluoroquinolone-resistant strains. Fluoroquinolones have also been associated with increased risks of dysglycemia, especially hyperglycemia, in patients with diabetes [11, 12]. The fluoroquinolone susceptibility test breakpoints of ciprofloxacin and levofloxacin to *Enterobacteriaceae* and *Pseudomonas aeruginosa* have been lowered by the Clinical and Laboratory Standards Institute (CLSI) in 2019 to help the detection of low-level fluoroquinolone resistance in these strains [12, 13]. By contrast, the prevalence of extended-spectrum β-lactamase (ESBL)-producing *E*. *coli* from UTIs, although much lower than the prevalence of fluoroquinolone-resistant ones, was estimated to exceed 15% in the USA and range 5–67% in the Asia-Pacific region [7, 9]. Beta-lactams are considered inferior to fluoroquinolones for the treatment of UTIs, despite most evidence being from outpatient settings [14].

Patients with diabetes have an increased risk for serious infections, which may in part be due to the higher prevalence of comorbidities and drug-resistant pathogens in patients with diabetes than those without [2, 15, 16]. Other mechanisms, including immune system dysfunction, glycosuria, and increased bacterial adherence to uroepithelial cells, may also explain why these patients have more severe and worse outcomes [17]. Diabetic patients have also been reported to have a higher risk of treatment failure of UTIs. [15, 18]. Despite the importance of selecting the most appropriate therapy for diabetic patients, there is limited evidence suggesting which empirical antimicrobial regimen may be a better choice for these patients.

This study aimed to investigate the effectiveness of β-lactams compared to fluoroquinolones as empirical therapy along with the clinical influence on the implementation of the new CLSI breakpoints for diabetic patients hospitalized for UTIs.

## Methods

### Study design and settings

We conducted a retrospective cohort study in diabetic patients aged 20 years or more hospitalized for symptomatic UTIs in Kaohsiung Medical University Hospital (KMUH), a tertiary medical center in southern Taiwan with approximately 1600 beds, during 2016–2018. The patients included had a positive urine culture, at least three outpatient type 2 diabetes diagnoses or an inpatient type 2 diabetes diagnoses [ICD-9-CM codes 250.xx (excluding 250.x1 or 250.x3), ICD-10-CM codes E11-E14] within one year before the admission to the general wards, inpatient UTI diagnosis, and at least one UTI symptom at admission. The UTI symptoms included fever, suprapubic tenderness, costovertebral angle tenderness, urinary urgency, urinary frequency, dysuria, and others such as general weakness or hematuria [19]. Positive urine cultures were defined as ≥10^5^ colony-forming units per milliliter (CFU/mL) of pathogens in midstream urine or Foley urine specimen, or ≥10^3^ CFU/mL of pathogens in simple catheterization, nephrostomy specimen, or suprapubic puncture.

Patients with any UTI diagnosis within one year before the admission, co-infections at the admission, ≥2 pathogens in the urine cultures, genitourinary defects, or intensive care unit (ICU) admission were excluded. The clinical characteristics, microbial etiology, and antibiograms of recurrent UTI can be different from those UTI events being not recurrent. Although recurrent UTI is usually defined as two or more UTIs within the last six months or three or more UTIs in the last 12 months [20], we adapted the definition to exclude patients with another UTI diagnosis within one year before the current UTI to exclude recurrent UTIs in the study.

The index date was defined as the admission date [21]. Comorbidities of the patients were traced back one year before the admission, and the Charlson comorbidity index (CCI) was calculated [22]. Quick sequential organ failure assessment (qSOFA) and sequential organ failure assessment (SOFA) scores were assessed for disease severity on admission [23, 24]. All patients were followed for at least 30 days after the admission unless the outcomes occurred. The primary outcome was empiric treatment failure, defined as the occurrence of any of the following events during the empirical treatment: a prescription of different UTI antimicrobial agents due to unresolved signs or symptoms, or death. The judgment of unresolved signs or symptoms was based on the physician’s note and lab data in the medical records.

The secondary outcomes included in-hospital mortality, 30-day mortality, the length of the stay, relapse within 30 days, and reinfection within 30 days. Relapse was defined as the same pathogen with an identical susceptibility profile in the urine cultures of the recurrent UTIs. Reinfection was defined as different isolated pathogens or different susceptibility profiles in the urine cultures of the recurrent UTIs. This study was approved by the Institutional Review Board of Kaohsiung Medical University Hospital (**KMUHIRB-E(II)-20190287**). Due to the retrospective nature of the study, informed consent was waived.

### Definitions

The identification and susceptibility testing interpretation of pathogens were performed by Kirby-Bauer disk diffusion test or Vitek 2 automated systems, according to CLSI criteria in 2018 and 2019 [25, 26]. Empirical antimicrobial therapy was defined as the first antimicrobial agent administered after the UTI diagnosis before the urine culture results. The β-lactams group included intravenous administration of cefazolin, cefmetazole, ceftriaxone, ceftazidime, cefoperazone/sulbactam, cefepime, flomoxef, ampicillin/sulbactam, piperacillin/tazobactam, and ertapenem. The fluoroquinolone groups included intravenous levofloxacin and ciprofloxacin. The empirical treatment was considered inappropriate if the pathogen was not susceptible to the administered agent.

Prior hospitalization was defined as any hospitalization within 30 days before the admission. Prior antimicrobial agent was defined as prescribed any antimicrobial agents within 14 days before the admission. Upper UTI was defined as a UTI with loin pain, flank tenderness, fever, rigors, and other manifestations of systemic inflammatory responses. Lower UTI was defined as a UTI with dysuria, urinary urgency, and urinary frequency without back pain, chills, or fever. Nosocomial UTI and community-acquired UTI were defined according to Aguilar-Duran and colleagues [19]. Bacteremic UTIs referred to the patients who also had positive blood culture without other identifiable sources of infections.

### Statistical analysis

Continuous variables were presented as medians and interquartile ranges (IQRs) and group differences were compared using the Mann-Whitney U test. Categorical variables were presented as numbers (N) and percentages (%) and group differences were compared using Fisher’s exact test.

For analyzing the association between the treatment and the outcomes, univariable and multivariable logistic regressions were utilized for categorical outcomes and a generalized linear model (GLM) with exponential family and reciprocal link function was utilized for continuous outcomes [27]. Variables significantly different between the β-lactams group and the fluoroquinolones group at baseline (Table 1) were adjusted in the multivariable models. To further elucidate the effect of the factors on treatment selection, sensitivity analyses varying the baseline characteristics, including the types of UTI, gender, comorbidities, renal function, hemoglobin A1c <8.0%, the susceptibility profile of the pathogens, as well as the appropriateness of the empirical therapy were performed. Hemoglobin A1c level of 8% was set as the cutoff level because this was the level recommended for older adults with multiple coexisting chronic illnesses [28], similar to this patient population.

**Table 1:** Patients characteristics.

Characteristics	All patients (N = 298)	β-lactams (N = 233)	Fluoroquinolones (N = 65)	P-value*
**Demographic**				
**Age**	76 (68.00–83.00)	77 (68.00–83.50)	71 (63.50–81.00)	**0.003**
**Gender (male)**	76 (25.50)	56 (24.03)	20 (30.77)	0.266
**Smoker**	32 (10.74)	24 (10.30)	8 (12.31)	0.653
**Alcohol**	18 (6.04)	13 (5.58)	5 (7.69)	0.557
**Upper UTI**	263 (88.26)	203 (87.12)	60 (92.31)	0.382
**Nosocomial UTI**	101 (33.89)	79 (33.91)	22 (33.85)	1.000
**Prior simple catheterization^a^**	32 (10.74)	25 (10.73)	7 (10.77)	1.000
**Prior foley^b^**	42 (14.09)	31 (13.30)	11 (16.92)	0.429
**Prior hospitalization^c^**	27 (9.06)	21 (9.07)	6 (9.23)	1.000
**Prior antimicrobial agent^d^**	13 (4.36)	12 (5.15)	1 (1.54)	0.311
**Comorbidity**				
**AMI^e^**	41 (13.76)	38 (16.31)	3 (4.62)	**0.014**
**Dementia**	36 (12.08)	32 (13.73)	4 (6.15)	0.131
**Liver disease**	28 (9.40)	23 (9.87)	5 (7.69)	0.810
**Renal disease**	88 (29.53)	70 (30.04)	18 (27.69)	0.761
**CHF**	24 (8.05)	21 (9.01)	3 (4.62)	0.311
**Pulmonary disease**	21 (7.05)	17 (7.30)	4 (6.15)	1.000
**Cancer**	60 (20.13)	45 (19.31)	15 (23.08)	0.489
**Diabetic complications**	226 (75.84)	177 (75.97)	49 (75.38)	1.000
**Cerebrovascular disease**	87 (29.19)	69 (29.61)	18 (27.69)	0.878
**Peptic ulcer**	70 (23.49)	56 (24.03)	14 (21.54)	0.743
**CCI**	3 (2.00–4.00)	3 (2.00–4.00)	3 (2.00–4.00)	0.103
**Patient Source**				
**Emergency room**	286 (95.97)	225 (96.57)	61 (93.85)	0.302
**Outpatient**	12 (4.03)	8 (3.43)	4 (6.15)	-
**On admission day**				
**qSOFA score**	0 (0.00–1.00)	0 (0.00–1.00)	0 (0.00–1.00)	0.252
**SOFA score**	2 (1.00–4.00)	2 (1.00–4.00)	3 (0.00–3.50)	0.334
**Bacteremic UTI**	110 (36.91)	84 (36.05)	26 (40.00)	0.564
**Temperature (°**C**)**	38.50 (38.00–39.20)	38.40 (38.00–39.10)	38.80 (38.40–39.40)	**0.011**
**Creatinine (mg/dL)**	1.14 (0.84–1.72)	1.17 (0.85–1.74)	1.01 (0.78–1.53)	0.194
**Clcr (mL/min/1.73m^2^)**	56.72 (36.94–79.28)	56.12 (35.90–78.42)	62.13 (45.27–82.11)	0.270
**C-reactive protein (mg/dL)**	60.18 (21.86–136.98)	59.59 (20.79–133.58)	60.76 (23.20–150.23)	0.761
**HbA1c (%)**	7.00 (6.20–8.40)	7.00 (6.20–8.50)	6.90 (6.20–7.55)	0.226
**Pathogens ^f^**				
***Acinetobacter baumannii***	1 (0.34)	1 (0.43)	0 (0.00)	1.000
***Citrobacter* species**	12 (4.03)	7 (3.00)	5 (7.69)	0.144
***Enterobacter* species**	6 (2.01)	6 (2.57)	0 (0.00)	0.346
***Escherichia coli***	182 (61.07)	139 (59.66)	43 (66.15)	0.389
***Klebsiella pneumoniae***	37 (12.41)	30 (12.88)	7 (10.77)	0.832
***Proteus mirabilis***	22 (7.38)	20 (8.58)	2 (3.08)	0.181
***Pseudomonas aeruginosa***	9 (3.02)	8 (3.43)	1 (1.54)	0.689
***Streptococcus* species**	6 (2.01)	5 (2.15)	1 (1.54)	1.000
**Others**	23 (7.73)	17 (7.30)	6 (9.23)	0.603
**Multidrug-resistant bacteria^g^**	60 (20.13)	46 (19.74)	14 (21.54)	0.750

Data were presented as N (%) or median (IQR).

UTI: Urinary tract infection; AMI: Acute myocardial infarction; CHF: Chronic heart failure; CCI: Charlson comorbidity index; qSOFA: quick sequential organ failure assessment; SOFA: sequential organ failure assessment; Clcr: Creatinine clearance

^*^P-value was calculated by Mann-Whitney U test or Fisher’s exact test as appropriate.

^a^Prior simple catheterization was defined as receiving simple catheterization before the admission

^b^Prior foley was defined as receiving foley before the admission

^**c**^Prior hospitalization was defined as hospitalization within 30 days before admission

^**d**^Prior antimicrobial agent was defined as receiving any antimicrobial agents within 14 days before admission

^e^AMI was defined as patients with acute myocardial infarction or old myocardial infarction, ICD codes (ICD-9: 410, 412; or ICD-10: I21, I22, I252), within one year before the admission.

^f^Citrobacter species was defined as Citrobacter freundii and Citrobacter koseri; Enterobacter species was defined as Enterobacter aerogenes, Enterobacter asburiae, and Enterobacter cloacae; Streptococcus species was defined as Streptococcus agalactiae, Streptococcus anginosus, Streptococcus gallolyticus and Streptococcus oralis. Others were defined as Morganella morganii, Providencia stuartii, Candida species (Candida albicans and Candida tropicalis), Enterococcus species (Enterococcus faecalis, Enterococcus faecium and Enterococcus hirae), Serratia species (Serratia marcescens and Serratia ureilytica), and Staphylococcus species (Staphylococcus aureus and Staphylococcus haemolyticus).

^g^ Multidrug-resistant bacteria was defined as non-susceptibility to at least one agent in three or more antimicrobial categories: penicillins, carbapenems, cephalosporins, aminoglycosides, and fluoroquinolones.

A 1:1 nearest neighbor propensity score matching without replacement was performed with a caliper width of 0.2. Patients in the β-lactams group and fluoroquinolones group were matched on the basis of all baseline variables as balanced as the standard mean difference (SMD) between ±0.25. The p-value in propensity score analysis was estimated by using conditional logistic regression. Two-tailed p-values less than 0.05 were considered statistically significant. All analyses in our study were performed by SPSS v.20.0 (IBM Corp., Armonk, NY, USA).

## Results

### Patient characteristics

**Fig 1:**
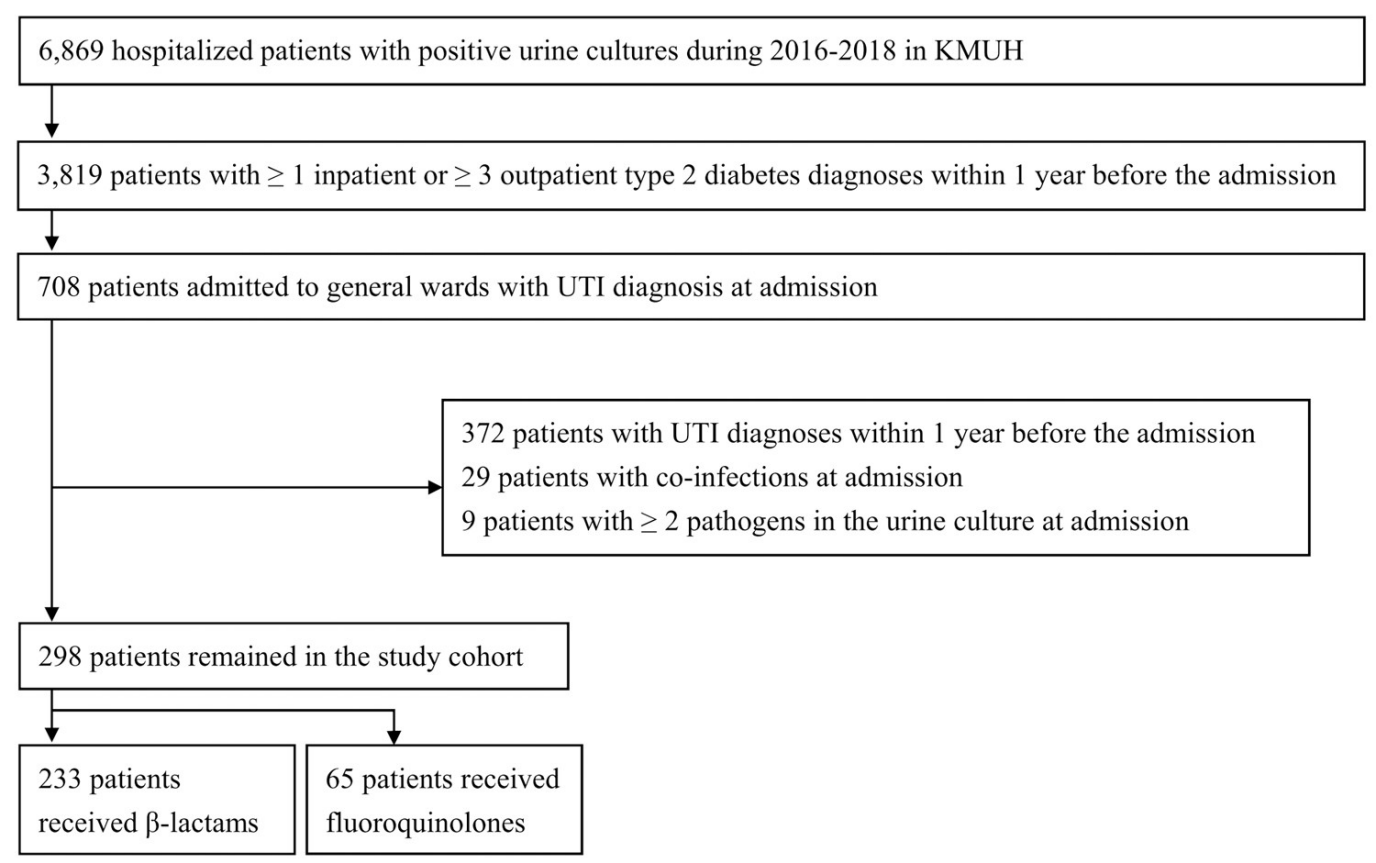
Flowchart of the study. KMUH: Kaohsiung Medical University Hospital; UTI: urinary tract infection.

From the 6,869 patients with positive urine cultures admitted to the hospital during the study period in KMUH, 3,819 patients were type 2 diabetes patients (Fig 1). Among these patients, 708 were diagnosed to have UTIs and admitted to general wards. After excluding patients with other UTI diagnoses within one year before admission, patients with co-infections at admission, and patients with more than one pathogen in the urine cultures, a total of 298 were included in our study. Based on the empirical treatment they received for the infection, there were 233 (78.19%) patients in the β-lactams group and 65 (21.81%) patients in the fluoroquinolones group.

The median age of the hospitalized UTI patients with type 2 diabetes was 76 years old and about 26% of them were male (Table 1). More than 88% (263/298) of all the patients had upper UTIs and about 34% (101/298) were nosocomial UTIs. About 37% (110/298) of the patients were bacteremic UTI. None of the patients were required to be transferred to ICU during the empiric antibiotic treatment.

There were no differences between the two groups in most baseline characteristics except age, acute myocardial infarction (AMI) history, and temperature on admission day. *E*. *coli* was the most common pathogen (61.07%), followed by *Klebsiella pneumoniae* (12.41%) and *Proteus mirabilis* (7.38%). There were 199 patients (66.78%), in which 156 (66.95%) in the β-lactams group and 43 (66.15%) in the fluoroquinolones group, switched to oral therapy when discharged to complete the course of antibiotic treatment.

The fluoroquinolone resistance rate, represented by levofloxacin (LEV) resistance, was 28.52% in all pathogens identified in this cohort and 31.32% in *E*. *coli* according to the pre-2019 CLSI interpretive criteria (S1 Table). The LEV resistance rate increased to 34.22% and 33.52% for all pathogens and *E*. *coli*, respectively, after applying the new 2019 CLSI interpretive criteria. The resistance rates of ceftazidime (CAZ) and cefepime (FEP) were 21.81% and 11.41%, respectively, in all pathogens identified in this cohort. The CAZ and FEP resistance rates in *E*. *coli* were 17.03% and 3.30%, respectively. According to the antimicrobial susceptibility testing, 181 (77.68%) patients received appropriate empiric antibiotics in the β-lactams group. In the fluoroquinolones group, there were 43 (66.15%) and 32 (49.23%) patients who received appropriate empiric therapy based on the pre-2019 CLSI interpretive criteria and 2019 CLSI interpretive criteria, respectively (S2 Table).

### Outcomes of the cohort in different empirical therapies

Since age, AMI, and temperature on admission were different between the two groups at baseline, the three factors were adjusted in all multivariable models. The β-lactams group was associated with less treatment failure compared to the fluoroquinolones group (adjusted OR = 0.32, 95% CI = 0.17–0.60) (Table 2) in this patient population. There was no significant difference between the β-lactams group and the fluoroquinolones group in in-hospital mortality, 30-day mortality, relapse within 30 days, reinfection within 30 days, and length of stay. We consistently found that the β-lactams group was associated with less treatment failure as compared with the fluoroquinolones group in the 126 propensity score-matched patients (OR 0.40, 95% CI 0.18–0.91, p = 0.028) (S3 Table).

**Table 2:** Outcomes of the cohort in different empirical therapies.

Outcomes	All patients (N = 298)	β-lactams (N = 233)	Fluoro-quinolones (N = 65)	Crude OR	P-value	Adjusted OR*	P-value
**Treatment failure**	103 (34.56)	67 (28.76)	36 (55.38)	0.32 (0.18–0.57)	**< .001**	0.32 (0.17–0.60)	**< .001**
**In-hospital mortality^a^**	9 (3.02)	8 (3.43)	1 (1.54)	2.27 (0.28–18.53)	0.396	1.26 (0.14–11.26)	0.837
**30-day mortality^a^**	8 (2.69)	7 (3.00)	1 (1.54)	1.98 (0.24–16.41)	0.492	0.84 (0.09–7.73)	0.878
**Relapse within 30 days**	5 (1.68)	4 (1.72)	1 (1.54)	1.40 (0.16–12.23)	0.759	1.13 (0.12–10.40)	0.912
**Reinfection within 30 days**	33 (11.07)	25 (10.73)	8 (12.31)	0.82 (0.35–1.92)	0.644	0.57 (0.21–1.53)	0.264
**Length of stay (days)^b^**	7 (5.00–10.25)	8 (5.00–10.50)	7 (5.50–10.50)	1.00 (0.98–1.01)	0.675	1.00 (0.98–1.01)	0.718

Data were presented as N (%) and median (IQR). We set the fluoroquinolones group as the reference group.

^*^Adjusted covariates: age, AMI, temperature

^a^ These cases requiring ICU care requested No-ICU-admission and Do-Not-Resuscitation.

^b^Length of stay (days) calculated by generalized linear model (GLM) with exponential family and reciprocal link function, other outcomes calculated by univariable and multivariable logistic regression.

Within the β-lactams group, the first-generation cephalosporin, cefazolin, as empirical antimicrobial therapy was not associated with less treatment failure (aOR = 1.97, 95% CI = 0.94–4.17) than non-cefazolin (S4 and S5 Tables).

### Sensitivity analyses of treatment failure

**Fig 2:**
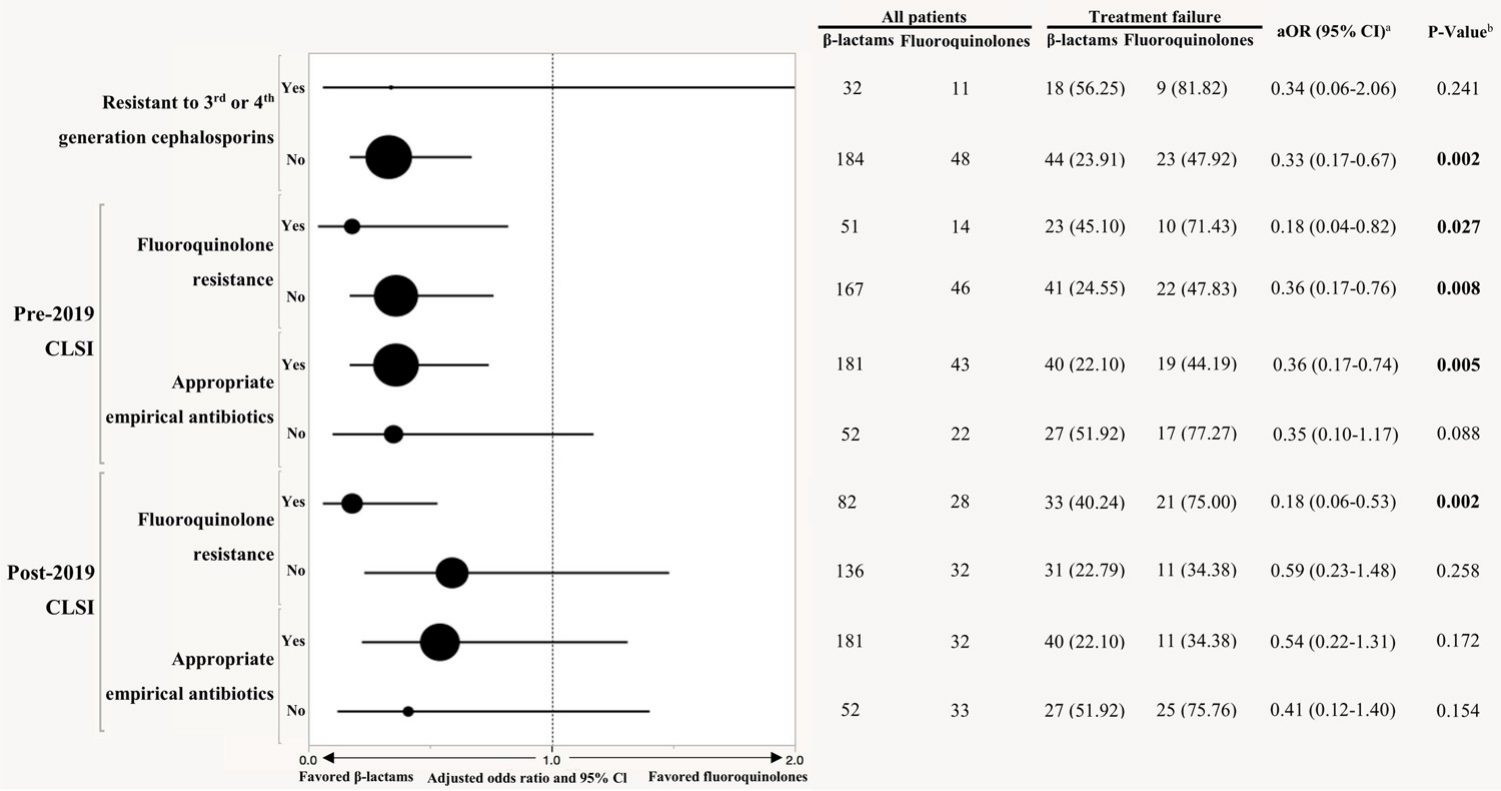
Forest plot of treatment failure by resistance patterns. Data were presented as N (%). The fluoroquinolones group was the reference group. The horizontal lines running through the dots represented the 95% CIs. The size of the dot for each aOR in the plot is proportional to the number of patients. UTI: Urinary tract infection; CCI: Charlson comorbidity index; Clcr: Creatinine clearance; SOFA: sequential organ failure assessment; Adjusted covariates in aOR: age, AMI, temperature. *P-value* was calculated by multivariable logistic regression.

To evaluate the effect of treatment due to susceptibility profile, we stratified the pathogens based on their 3rd or 4th generation cephalosporin resistance, fluoroquinolone resistance, and the appropriateness of the empirical therapy (Fig 2). When pathogens were susceptible to 3rd or 4th generation cephalosporins, the β-lactam group was associated with less treatment failure (aOR = 0.33, 95% CI = 0.17–0.67). The β-lactam group was also associated with less treatment failure than the fluoroquinolone group in pathogens not resistant to fluoroquinolones as defined by pre-2019 CLSI breakpoints (aOR = 0.36, 95% CI = 0.17–0.76). Among those who received effective empirical antimicrobial agents as defined by the pre-2019 breakpoint, the β-lactam group was associated with less treatment failure than the fluoroquinolones group (aOR = 0.36, 95% CI = 0.17–0.74). No significant difference in treatment failure was found in pathogens not resistant to fluoroquinolones as defined by 2019 CLSI breakpoints (aOR = 0.59, 95% CI = 0.23–1.48). Among those who received effective empirical antimicrobial agents as defined by 2019 CLSI breakpoints, no significant difference in treatment failure was found (aOR = 0.54, 95% CI = 0.22–1.31).

**Fig 3:**
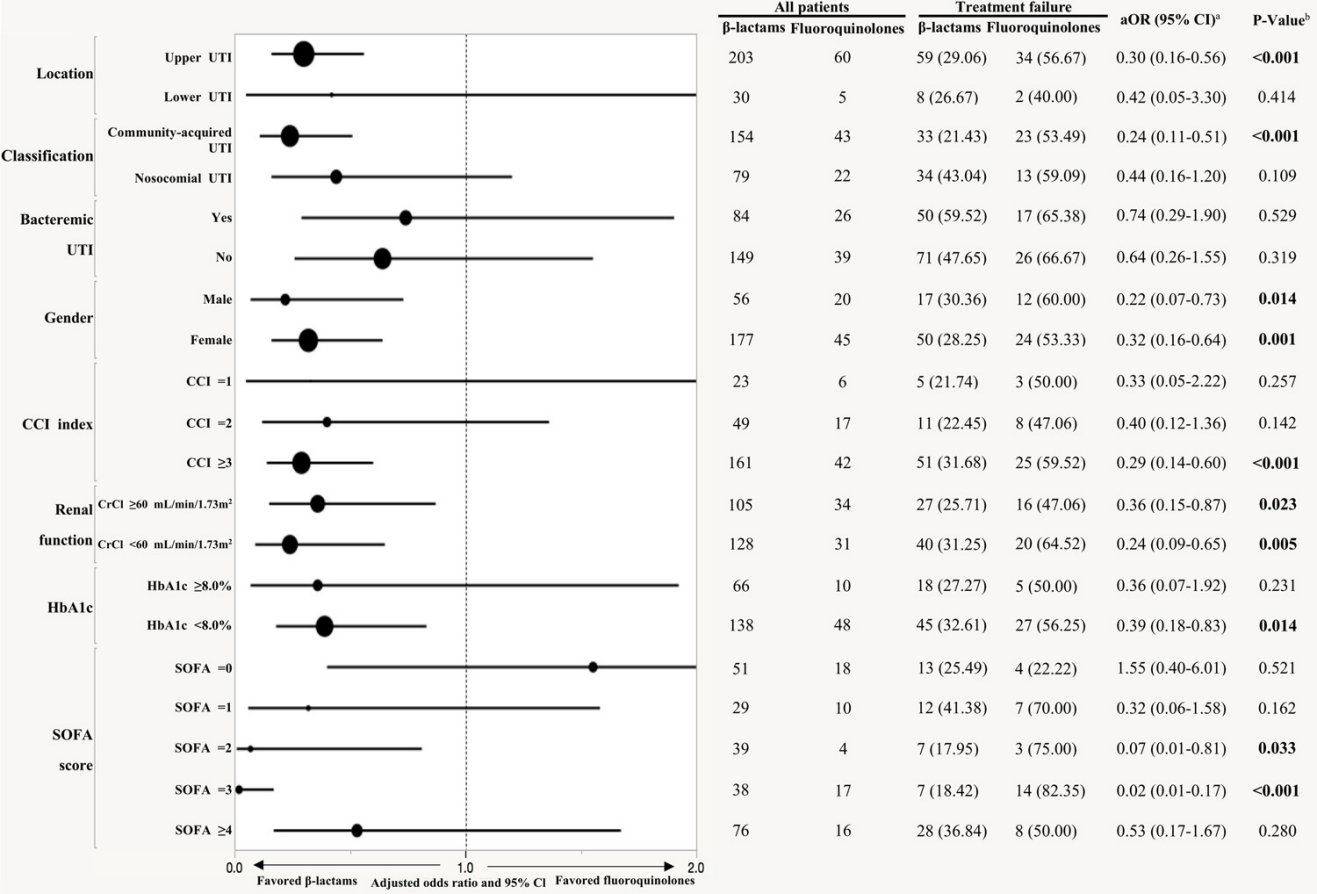
Forest plot of treatment failure by patient characteristics. Data were presented as N (%). The fluoroquinolones group was the reference group. The horizontal lines running through the dots represented the 95% CIs. The size of the dot for each aOR in the plot is proportional to the number of patients. UTI: Urinary tract infection; CCI: Charlson comorbidity index; Clcr: Creatinine clearance; SOFA: sequential organ failure assessment; Adjusted covariates in aOR: age, AMI, temperature. *P-value* was calculated by multivariable logistic regression.

The β-lactams group, compared to the fluoroquinolones group, was associated with less treatment failure in upper UTIs (aOR = 0.30, 95% CI = 0.16–0.56), community-acquired UTIs (aOR = 0.24, 95% CI = 0.11–0.51), CCI ≥3 (aOR-1 = 0.29, 95% CI = 0.14–0.60), HbA1c <8.0% (<64 mmol/mol, aOR = 0.39, 95% CI = 0.18–0.83), and SOFA score = 2 or 3 (aOR = 0.07, 95% CI = 0.01–0.81; aOR = 0.02, 95% CI = 0.01–0.17) (Fig 3). There was no difference in the rates of bacteremia due to UTI between the two treatment groups. The β-lactams group was associated with less treatment failure than the fluoroquinolones group regardless of gender and renal function.

## Discussion

We conducted a retrospective cohort study for suitable empirical antimicrobial therapy for patients with type 2 diabetes hospitalized for UTIs. Beta-lactams as empirical antimicrobial agents were associated with less treatment failure than fluoroquinolones, in pathogens either resistant to fluoroquinolones or susceptible to 3rd or 4th generation cephalosporins. Beta-lactams were associated with less treatment failure than fluoroquinolones only when the appropriateness of the empirical therapy was determined by the pre-2019 CLSI breakpoints. Patients with upper UTIs, community-acquired UTIs, comorbidity score ≥ 3, HbA1c less than 8.0% (64 mmol/mol), or SOFA score 2 or 3, maybe more likely to benefit from β-lactams as empirical antimicrobial agents. This study provided clinical evidence to support the new 2019 CLSI breakpoint for fluoroquinolones as a good indicator for treatment outcomes in UTIs. Based on our search, this also appears to be the first study that evaluated the effectiveness of empirical therapies in hospitalized UTI patients with type 2 diabetes.

Based on the pre-2019 CLSI breakpoints, the resistance rates of *E*. *coli* reported in this study were 31% and 17% for levofloxacin and ceftazidime, respectively. A previous study analyzing drug-resistant patterns in urinary-tract-related *E*. *coli* from diabetic patients in southern Taiwan showed the fluoroquinolone-resistance and the 3rd-generation cephalosporin-resistance rates were 35% and 24%, respectively [2]. Among UTI pathogens in the US, the fluoroquinolone-resistance rate in *E*. *coli* was reported to be around 32%, and the 3rd- and 4th-generation cephalosporin-resistant *E*. *coli* was approaching 5% between 2007 and 2010 [29]. In Europe, resistance to fluoroquinolones in *E*. *coli* in UTIs varies by country, ranging from 1.6% to 13%, and ESBL was confirmed in around 2% of *E*. *coli* strains [30]. Given the high resistance rates in our area, neither fluoroquinolones nor β-lactams should be recommended as empirical therapy for these patients. In line with the relatively higher fluoroquinolone resistance rate than β-lactams resistance rates, we found that β-lactams were associated with less treatment failure rate than fluoroquinolones.

By applying the revised 2019 CLSI breakpoints with levofloxacin MICs ≥2 μg/mL, there was about a 2% increase in the overall fluoroquinolone resistance rate in comparison to the pre-2019 CLSI breakpoints with levofloxacin MICs ≥8 μg/mL in our study. The 2019 CLSI fluoroquinolones breakpoint revisions for *Enterobacteriaceae* and *P*. *aeruginosa* were based on PK/PD models rather than clinical observations [11]. Yet, the breakpoint change made the empirical failure rates of fluoroquinolones as empirical therapy compatible with β-lactams in susceptible strains. Levofloxacin was shown to result in higher mortality in patients with *Enterobacterales* bacteremia infected by isolates with intermediate levofloxacin susceptibility (MIC 1 or 2μg/mL) than those susceptible to levofloxacin (MIC ≤0.5 μg/mL) [31]. In concordance with the previous study, our results supported that the revised fluoroquinolone breakpoints predict clinical outcomes well.

Cefazolin, a first-generation cephalosporin in an area with an acceptable resistance rate in *E*. *coli*, has been considered non-inferior to fluoroquinolones for the treatment of community-acquired UTIs in hospitalized patients [32]. Although not significant, cefazolin trended to be associated with an increased treatment failure rate than non-cefazolin. The overall cefazoline resistance rate was about 34% in our population.

There were several strengths in our study. First, we were able to use the persistence of UTI symptoms as an indicator for empiric treatment failure. Second, we were also able to evaluate the impact of diabetes control (HbA1c level) and renal functions (CrCl) of the patients on medication selection. Third, all patients in our study were UTIs requiring hospitalization and intravenous antimicrobial therapy. The patient population was relatively homogeneous as patients with genitourinary defects and recurrent UTIs were excluded.

There were some limitations in our study. First, given the retrospective nature of the study, some confounders might be missing. For example, the urobiome may also play a role in shaping the bacterial infection and the response to antimicrobial therapy in this patient population, but we also were not able to evaluate this part in this study. There may also be bias-by-indication and other confounding factors. To minimize the confounding effects and the potential bias, we incorporated all variables which may affect treatment effects, including demographic characteristics, comorbidities, and lab data from the medical records in our study. Our results from multivariable logistic regression models and propensity score matching were consistent. Second, our secondary outcomes, including in-hospital mortality, 30-day mortality, and recurrence rate, were likely influenced by the definitive therapies. In addition, the event rates of the secondary outcomes were low. Conclusive conclusions of the secondary outcomes between the treatment groups cannot be made. Third, the sample size was not large enough to compare treatment differences between subgroups. Our sensitivity analyses suggested that some populations may especially benefit from β-lactams as empirical therapy. Yet, no differences between the subgroups were observed due to wide confidence intervals. Finally, the results may not be extrapolated to patients requiring intensive care, patients with recurrent UTIs, or patients in hospitals with different antibiograms. Excluding patients who were admitted to ICU during the empiric antibiotic treatment do limit the application of our findings to the non-ICU case group yet ensures that the patients in both treatment groups have comparable severity during the empiric treatment period.

In conclusion, β-lactams are suitable empirical therapy over fluoroquinolones in type 2 diabetes patients admitted due to UTIs in areas with a high prevalence of fluoroquinolone resistance. The new 2019 CLSI breakpoint for fluoroquinolone correlate with treatment outcome well in this population.

## Supporting information

S1 Data (XLSX)

S1 Table Resistance patterns of pathogens in the cohort. (DOCX)

S2 Table The proportion of patients who received appropriate empiric therapy according to the antimicrobial susceptibility testing report. (DOCX)

S3 Table Baseline characteristics and outcome of 126 patients after 1:1 propensity score matching. (DOCX)

S4 Table Cefazolin vs. non-cefazolin baseline. (DOCX)

S5 Table Cefazolin vs. non-cefazolin primary outcome. (DOCX)
