# Supplementary material for: Clinical effectiveness of beta-lactams versus fluoroquinolones as empirical therapy in patients with diabetes mellitus hospitalized for urinary tract infections: A retrospective cohort study
Source: PLoS One. 2022 Mar 31;17(3):e0266416. doi: 10.1371/journal.pone.0266416 (PMC8970481; doi:10.1371/journal.pone.0266416)
Supplement: S1 Table — (DOCX) [file pone.0266416.s002.docx]

**S1 Table.** **Resistance patterns of pathogens in the cohort.**

| Organisms isolated^a^ |  | Resistance pattern (% resistance) | | | | | | | | | | |
| --- | --- | --- | --- | --- | --- | --- | --- | --- | --- | --- | --- | --- |
| Antimicrobial agents | AM  (N=268) | SAM  (N=269) | SXT  (N=269) | CZ  (N=268) | LEV^b^  (N=278) | LEV^c^  (N=278) | GM  (N=275) | CAZ  (N=275) | TGC  (N=280) | CMZ  (N=265) | FEP  (N=275) | TZP  (N=275) |
| *Acinetobacter baumannii* (N=1) | N/A | 100.00 | 100.00 | N/A | 0.00 | 100.00 | 100.00 | 100.00 | 100.00 | N/A | 100.00 | 100.00 |
| *Candida* species (N=3) | N/A | N/A | N/A | N/A | N/A | N/A | N/A | N/A | N/A | N/A | N/A | N/A |
| *Citrobacter* species (N=12) | 100.00 | 91.66 | 8.33 | 25.00 | 0.00 | 8.33 | 0.00 | 8.33 | 0.00 | 25.00 | 0.00 | 0.00 |
| *Enterobacter* species (N=6) | 100.00 | 100.00 | 33.33 | 100.00 | 16.67 | 16.67 | 0.00 | 16.67 | 0.00 | 100.00 | 0.00 | 16.67 |
| *Enterococcus* species (N=11) | N/A | N/A | N/A | N/A | N/A | N/A | N/A | N/A | 0.00 | N/A | N/A | N/A |
| *Escherichia coli* (N=182) | 68.68 | 34.07 | 36.26 | 25.27 | 31.32 | 33.52 | 24.73 | 17.03 | 0.00 | 4.95 | 3.30 | 3.85 |
| *Klebsiella pneumoniae* (N=37) | 100.00 | 21.62 | 24.32 | 21.62 | 13.51 | 16.21 | 18.91 | 16.21 | 5.41 | 0.00 | 10.81 | 5.41 |
| *Morganella morganii* (N=2) | 100.00 | 50.00 | 0.00 | 100.00 | 0.00 | 0.00 | 0.00 | 0.00 | 100.00 | 0.00 | 0.00 | 0.00 |
| *Proteus mirabilis* (N=22) | 45.45 | 18.18 | 50.00 | 9.09 | 4.55 | 22.73 | 18.18 | 4.55 | 100.00 | 4.55 | 0.00 | 0.00 |
| *Providencia stuartii* (N=1) | 100.00 | 100.00 | 100.00 | 100.00 | 0.00 | 100.00 | 100.00 | 0.00 | 100.00 | 0.00 | 0.00 | 0.00 |
| *Pseudomonas aeruginosa* (N=9) | N/A | N/A | N/A | N/A | 11.11 | 33.33 | 11.11 | 11.11 | N/A | N/A | 0.00 | 0.00 |
| *Serratia* species (N=3) | 100.00 | 100.00 | 33.33 | 100.00 | 0.00 | 0.00 | 33.33 | 0.00 | 0.00 | 0.00 | 0.00 | 0.00 |
| *Staphylococcus* species (N=3) | N/A | 33.33 | 0.00 | 33.33 | N/A | N/A | N/A | N/A | 0.00 | N/A | N/A | N/A |
| *Streptococcus* species^d^ (N=6) | 0.00 | N/A | N/A | N/A | 100.00 | 100.00 | N/A | N/A | N/A | N/A | N/A | N/A |
| Overall resistance^e^ (%) | 75.84 | 42.62 | 40.60 | 34.23 | 28.52 | 34.22 | 27.85 | 21.81 | 15.10 | 17.45 | 11.41 | 11.41 |

Data were presented as percentages.

N/A: Not available; AM: Ampicillin; SAM: Ampicillin/Sulbactam; SXT: Sulfamethoxazole/Trimethoprim; CZ: Cefazolin; LEV: Levofloxacin; GM: Gentamicin; CAZ: Ceftazidime; TGC: Tigecycline; CMZ: Cefmetazole; FEP: Cefepime; TZP: Piperacillin/Tazobactam;

^a^*Candida* species was defined as *Candida* *albicans* and *Candida* *tropicalis*; *Citrobacter* species was defined as *Citrobacter* *freundii* and *Citrobacter* *koseri*; *Enterobacter* species was defined as *Enterobacter* *aerogenes*, *Enterobacter* *asburiae* and *Enterobacter* *cloacae*; *Enterococcus* species was defined as *Enterococcus* *faecalis*, *Enterococcus* *faecium* and *Enterococcus* *hirae*; *Serratia* species was defined as *Serratia* *marcescens* and *Serratia* *ureilytica*; *Staphylococcus* species was defined as *Staphylococcus* *aureus* and *Staphylococcus* *haemolyticus*; *Streptococcus* species was defined as *Streptococcus* *agalactiae*, *Streptococcus* *anginosus*, *Streptococcus* *gallolyticus* and *Streptococcus* *oralis.*

^b^ Pre-2019 CLSI definition for levofloxacin resistance;

^c^ Post-2019 CLSI definition for levofloxacin resistance.

^d^ Only 3 isolates had susceptibility data

^e^ Calculated by divided the total number of resistant strains by the total number of strains (n=298). Strains without susceptibility data were considered as resistant.
