# Supplementary material for: Clinical effectiveness of beta-lactams versus fluoroquinolones as empirical therapy in patients with diabetes mellitus hospitalized for urinary tract infections: A retrospective cohort study
Source: PLoS One. 2022 Mar 31;17(3):e0266416. doi: 10.1371/journal.pone.0266416 (PMC8970481; doi:10.1371/journal.pone.0266416)
Supplement: S2 Table — (DOCX) [file pone.0266416.s003.docx]

**S2 Table.** **The proportion of patients who received appropriate empiric therapy according to the antimicrobial susceptibility testing report.**

| **Appropriate empiric therapy** | | | | | |
| --- | --- | --- | --- | --- | --- |
| **β-lactams** (N=233) | n/N (%) |  | **Fluoroquinolones** (N=65) | Pre-2019 CLSI criteria n/N (%) | Post-2019 CLSI criteria n/N (%) |
| **1^st^ cephalosporin** | 28/37 (75.68) |  | Ciprofloxacin | 4/8 (50.00) | 3/8 (37.50) |
| Cefazolin | 28/36 (77.78) |  | Levofloxacin | 39/57 (68.42) | 29/57 (50.88) |
| Cephradine | 0/1 (0.00) |  | **Total** | 43/65 (66.15) | 32/65 (49.23) |
| **2^nd^ cephalosporin** | 84/107 (78.50) |  |  |  |  |
| Cefmetazole | 56/68 (82.35) |  |  |  |  |
| Cefuroxime | 7/10 (70.00) |  |  |  |  |
| Flomoxef | 21/28 (75.00) |  |  |  |  |
| Cefaclor | 0/1 (0.00) |  |  |  |  |
| **3^rd^ or 4^th^ cephalosporin** | 29/34 (85.29) |  |  |  |  |
| Ceftazidime | 6/6 (100.00) |  |  |  |  |
| Ceftriaxone | 20/24 (83.33) |  |  |  |  |
| Cefoperazone/sulbactam | 2/3 (66.67) |  |  |  |  |
| Cefepime | 1/1 (100.00) |  |  |  |  |
| **Penicillins /**  **β-lactamase inhibitors** | 15/27 (55.56) |  |  |  |  |
| Amoxicillin /clavulanate | 1/4 (25.00) |  |  |  |  |
| Ampicillin /sulbactam | 2/8 (25.00) |  |  |  |  |
| Piperacillin /tazobactam | 12/15 (80.00) |  |  |  |  |
| **Carbapenem** |  |  |  |  |  |
| Ertapenem | 25/28 (89.29) |  |  |  |  |
| **Total** | 181/233 (77.68) |  |  |  |  |
