# Supplementary material for: Clinical effectiveness of beta-lactams versus fluoroquinolones as empirical therapy in patients with diabetes mellitus hospitalized for urinary tract infections: A retrospective cohort study
Source: PLoS One. 2022 Mar 31;17(3):e0266416. doi: 10.1371/journal.pone.0266416 (PMC8970481; doi:10.1371/journal.pone.0266416)
Supplement: S3 Table — (DOCX) [file pone.0266416.s004.docx]

**S3 Table.** **Baseline characteristics and outcome of 126 patients after 1:1 propensity score matching.**

| **Characteristics** | All patients | β-lactams | Fluoroquinolones | Standard mean difference |
| --- | --- | --- | --- | --- |
|  | (N=126) | (N=63) | (N=63) |  |
| **Demographic** |  |  |  |  |
| Age | 71.0 (64.8-81.0) | 71.0 (64.0-80.0) | 72.0 (65.0-81.0) | -0.003 |
| Gender (male) | 38 (30.16) | 20 (31.75) | 18 (28.57) | 0.074 |
| Smoker | 16 (12.70) | 8 (12.70) | 8 (12.70) | 0.000 |
| Alcoholism | 10 (7.94) | 5 (7.94) | 5 (7.94) | 0.000 |
| Upper UTI | 112 (92.06) | 58 (92.06) | 58 (92.06) | 0.000 |
| Nosocomial UTI | 43 (34.13) | 22 (34.92) | 21 (33.33) | 0.033 |
| Prior simple catheterization | 16 (12.70) | 9 (14.29) | 7 (11.11) | 0.102 |
| Prior foley | 26 (20.63) | 15 (23.81) | 111 (17.46) | 0.187 |
| Prior hospitalization | 12 (9.52) | 6 (9.52) | 6 (9.52) | 0.000 |
| Prior antimicrobial agent | 2 (1.59) | 1 (1.59) | 1 (1.59) | 0.000 |
| **Comorbidity** |  |  |  |  |
| AMI | 9 (7.14) | 6 (9.52) | 3 (4.76) | 0.129 |
| Dementia | 8 (6.35) | 4 (6.35) | 4 (6.35) | 0.000 |
| Liver disease | 7 (5.56) | 2 (3.17) | 5 (7.94) | -0.159 |
| Renal disease | 35 (27.78) | 17 (26.98) | 18 (28.57) | -0.035 |
| CHF | 6 (4.76) | 3 (4.76) | 3 (4.76) | 0.000 |
| Pulmonary disease | 7 (5.56) | 3 (4.76) | 4 (6.35) | -0.061 |
| Cancer | 32 (25.40) | 17 (26.98) | 15 (23.81) | 0.080 |
| Diabetic complications | 96 (76.19) | 49 (77.78) | 47 (74.60) | 0.074 |
| Cerebrovascular disease | 35 (27.78) | 18 (28.57) | 17 (26.98) | 0.035 |
| Peptic ulcer | 30 (23.81) | 16 (25.40) | 14 (22.22) | 0.074 |
| CCI | 3.0 (2.0-4.0) | 3.0 (2.0-4.0) | 3.0 (2.0-4.0) | 0.063 |
| **Patient Source** |  |  |  |  |
| Emergency room | 117 (92.86) | 58 (92.06) | 59 (93.65) | -0.087 |
| Outpatient | 9 (7.14) | 5 (7.94) | 4 (6.35) | 0.087 |
| **On Admission day** |  |  |  |  |
| qSOFA score | 0 (0.00-1.00) | 0 (0.00-1.00) | 0 (0.00-1.00) | 0.098 |
| SOFA score | 2 (0.00-4.00) | 2 (1.00-4.00) | 3 (0.00-3.00) | 0.070 |
| Temperature (℃) | 38.50 (37.80-39.10) | 38.40 (37.70-39.00) | 38.70 (38.10-39.20) | 0.068 |
| Creatinine (mg/dL) | 1.10 (0.79-1.62) | 1.18 (0.79-1.72) | 1.03 (0.78-1.53) | -0.072 |
| Clcr (mL/min/1.73m^2^) | 56.57 (39.71-80.45) | 56.06 (39.18-80.09) | 61.72 (43.55-81.52) | -0.019 |
| C-reactive protein (mg/dL) | 61.67 (21.82-153.53) | 64.36 (20.72-167.36) | 58.5 (22.19-148.85) | 0.143 |
| HbA1c (%) | 6.75 (5.90-7.70) | 6.70 (5.90-8.10) | 6.90 (6.00-7.50) | 0.073 |
| **Outcome** |  |  |  | **P-value** |
| Empiric treatment failure | 58 (46.02) | 22 (34.92) | 36 (57.14) | 0.028 |
