# Supplementary material for: Clinical effectiveness of beta-lactams versus fluoroquinolones as empirical therapy in patients with diabetes mellitus hospitalized for urinary tract infections: A retrospective cohort study
Source: PLoS One. 2022 Mar 31;17(3):e0266416. doi: 10.1371/journal.pone.0266416 (PMC8970481; doi:10.1371/journal.pone.0266416)
Supplement: S4 Table — (DOCX) [file pone.0266416.s005.docx]

**S4 Table.** **Cefazolin vs. non-cefazolin baseline.**

| **Characteristics** | **All beta-lactams** | **Cefazolin** | **Non-cefazolin** | **P-value** |
| --- | --- | --- | --- | --- |
|  | **(N=233)** | **(N=38)** | **(N=195)** |  |
| **Demographic** |  |  |  |  |
| Age | 77.0 (68.0-83.5) | 76.5 (68.0-79.0) | 77.0 (68.0-84.0) | 0.297 |
| Gender (male) | 56 (24.03) | 6 (15.79) | 50 (25.64) | 0.292 |
| Smoker | 24 (10.30) | 2 (5.26) | 22 (11.28) | 0.385 |
| Alcoholism | 13 (5.58) | 3 (7.89) | 10 (5.13) | 0.450 |
| Upper UTI | 203 (87.12) | 31 (81.58) | 172 (88.21) | 0.290 |
| Nosocomial UTI | 79 (33.91) | 9 (23.68) | 70 (35.90) | 0.190 |
| Prior simple catheterization | 25 (10.73) | 5 (13.16) | 20 (10.26) | 0.572 |
| Prior foley | 31 (13.31) | 0 (0.00) | 31 (15.90) | **0.004** |
| Prior hospitalization | 21 (9.01) | 3 (7.89) | 18 (9.23) | 1.000 |
| Prior antimicrobial agent | 12 (5.15) | 0 (0.00) | 12 (6.15) | 0.224 |
| **Comorbidity** |  |  |  |  |
| AMI | 38 (16.31) | 7 (18.42) | 31 (15.90) | 0.640 |
| Dementia | 32 (13.73) | 6 (15.79) | 26 (13.33) | 0.617 |
| Liver disease | 23 (9.87) | 3 (7.89) | 20 (10.26) | 1.000 |
| Renal disease | 70 (30.04) | 10 (26.32) | 60 (30.77) | 0.700 |
| CHF | 21 (9.01) | 3 (7.89) | 18 (9.23) | 1.000 |
| Pulmonary disease | 17 (7.30) | 3 (7.89) | 14 (7.18) | 0.745 |
| Cancer | 45 (19.31) | 5 (13.16) | 40 (20.51) | 0.373 |
| Diabetic complications | 177 (75.97) | 27 (71.05) | 150 (76.92) | 0.416 |
| Cerebrovascular disease | 69 (29.61) | 12 (31.58) | 57 (29.23) | 0.846 |
| Peptic ulcer | 56 (24.03) | 9 (23.68) | 47 (24.10) | 1.000 |
| CCI | 3.0 (2.0-4.0) | 3.0 (2.0-5.0) | 3.0 (2.0-4.0) | 0.738 |
| **Patient Source** |  |  |  |  |
| Emergency room | 225 (96.57) | 36 (94.74) | 189 (96.92) | 0.620 |
| Outpatient | 8 (3.43) | 2 (5.26) | 6 (3.08) | - |
| **On Admission day** |  |  |  |  |
| Temperature (℃) | 38.40 (38.00-39.10) | 38.30 (37.60-39.00) | 38.50 (38.00-39.20) | 0.369 |
| Creatinine (mg/dL) | 1.17 (0.85-1.74) | 1.29 (0.92-1.55) | 1.14 (0.84-1.80) | 0.512 |
| Clcr (mL/min/1.73m^2^) | 56.12 (35.90-78.42) | 56.45 (39.67-69.92) | 56.12 (32.75-79.41) | 0.734 |
| C-reactive protein (mg/dL) | 59.59 (20.79-133.58) | 31.69 (13.02-72.29) | 65.14 (22.27-138.78) | **0.020** |
| HbA1c (%) | 7.00 (6.20-8.50) | 6.60 (6.00-7.95) | 7.10 (6.20-8.60) | 0.689 |
