# Supplementary material for: Clinical effectiveness of beta-lactams versus fluoroquinolones as empirical therapy in patients with diabetes mellitus hospitalized for urinary tract infections: A retrospective cohort study
Source: PLoS One. 2022 Mar 31;17(3):e0266416. doi: 10.1371/journal.pone.0266416 (PMC8970481; doi:10.1371/journal.pone.0266416)
Supplement: S5 Table — (DOCX) [file pone.0266416.s006.docx]

**S5 Table.** **Cefazolin vs. non-cefazolin primary outcome.**

| **Primary outcome** | **All beta-lactams** | **Cefazolin** | **Non-cefazolin** | **Crude OR** | **P-value** | **Adjusted OR** | **P-value** |
| --- | --- | --- | --- | --- | --- | --- | --- |
|  | **(N=233)** | **(N=38)** | **(N=195)** |  |  |  |  |
| Empiric treatment failure | 67 (28.76) | 15 (39.47) | 52 (26.67) | 1.79 (0.87-3.70) | 0.114 | 1.97 (0.94-4.17) | 0.074 |
| **-- Nosocomial UTI** | 34/79 (43.04) | 5/9 (55.56) | 29/70 (41.43) | 1.77 (0.44-7.15) | 0.425 | 2.35 (0.54-10.24) | 0.255 |
| **-- Community acquired UTI** | 33/154 (21.43) | 10/29 (34.48) | 23/125 (18.40) | 2.33 (0.96-5.68) | 0.062 | 2.36 (0.95-5.88) | 0.064 |

Adjusted factors: Prior foley, C-reactive protein
